# Equivalence of the GeneXpert System and GeneXpert Omni System for tuberculosis and rifampicin resistance detection

**DOI:** 10.1371/journal.pone.0261442

**Published:** 2021-12-17

**Authors:** Sophia B. Georghiou, Riccardo Alagna, Daniela M. Cirillo, Sergio Carmona, Morten Ruhwald, Samuel G. Schumacher

**Affiliations:** 1 FIND, The Global Alliance for Diagnostics, Geneva, Switzerland; 2 IRCCS San Raffaele Scientific Institute, Milan, Italy; Rutgers University, UNITED STATES

## Abstract

A laboratory validation study was conducted to assess the equivalence of Xpert MTB/RIF Ultra testing on the GeneXpert System and the GeneXpert Omni System (‘Omni’) for tuberculosis and rifampicin resistance. High concordance of the two devices was demonstrated for well-characterized clinical samples as well as control materials, with controls tested on Omni at normal and challenging environmental conditions (i.e. 35°C, 90% relative humidity). Equivalence of the Cts for all probes was also shown. Equivalence was demonstrated for the Omni and GeneXpert devices for tuberculosis and rifampicin resistance detection for a diverse range of clinical specimens and environmental conditions.

## Introduction

Despite the gains made in tuberculosis (TB) and drug-resistant TB (DR-TB) diagnosis since the launch and rollout of rapid molecular assays, including the Cepheid Xpert and Molbio TrueNat assays [1], diagnostic gaps remain. In 2019, 71% of the estimated 10 million people who developed TB were diagnosed, and only 61% of people with bacteriologically confirmed TB were tested for rifampicin (RIF) resistance [2]. The COVID-19 pandemic has further negatively impacted access to TB diagnosis and treatment around the globe and underscored the crucial need for point-of-care TB and DR-TB testing, especially at lower level healthcare centers where most patients enter the care cascade [3].

The use of rapid molecular assays in peripheral settings is limited by insufficient infrastructure (i.e. stable power supply) and challenging environmental conditions [4]. To address this issue, Cepheid has developed the GeneXpert Omni System (‘Omni’) for Xpert cartridge testing in remote, low-throughput settings with limited infrastructure. The Omni is a mobile-phone operated, single module, battery-powered, point-of-care device with cloud-based connectivity for data transfer and increased stability to dust, humidity and high temperatures [5]. To support policy and implementation, manufacturer-independent equivalence comparisons of Omni to GeneXpert devices are needed. We evaluated the performance of Xpert MTB/RIF Ultra (‘MTB Ultra’) when run on Omni compared to GeneXpert, including evaluation of Omni under environmental stressors (e.g. high temperature and high humidity) to determine whether the device can be used at point-of-care while maintaining equivalent performance to GeneXpert.

## Materials and methods

**Fig 1:**
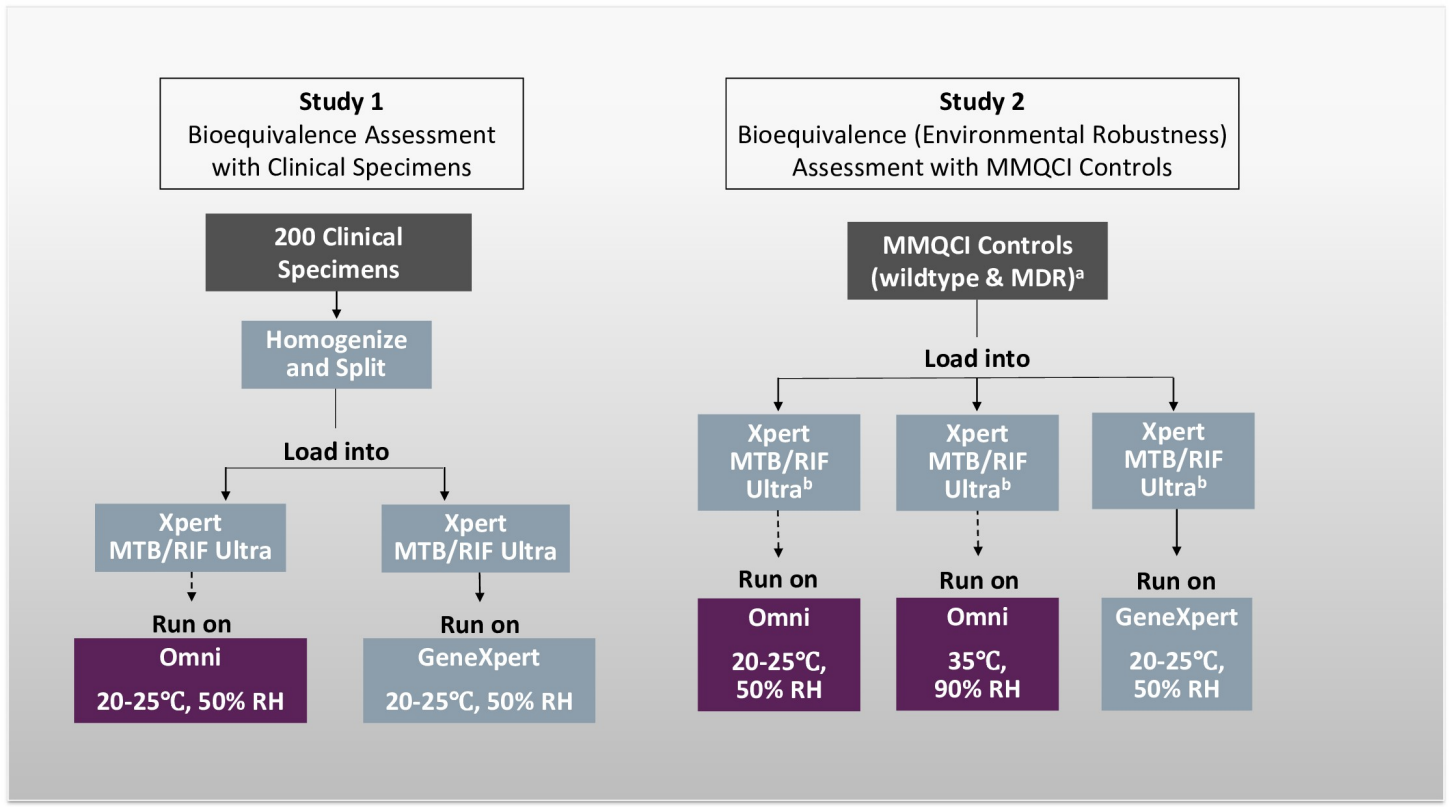
Omni bioequivalence study flow. RH: relative humidity, MDR: multidrug-resistant tuberculosis, MMQCI: Maine Molecular Quality Controls, SR: sample reagent. ^a^ 30 replicates of each control were tested by each device at the listed conditions (i.e. 30 replicates per condition) at 3x the limit of detection. Replicate testing was divided between 8 Omni devices and 6 modules of two GeneXpert-IV devices. Daily negative control testing was also performed for Omni and GeneXpert. ^b^ Ultra cartridges were stored at the respective condition (20–25°C, 50% RH or 35C, 90% RH) for 24hr prior to testing.

This manufacturer-independent laboratory assessment was carried out at the TB Supranational Reference Laboratory at the San Raffaele Scientific Institute, Milan, Italy. The full protocol is available in S1 File and study flow is shown in Fig 1. ‘Study 1’ used banked clinical specimens to assess the positive and negative concordance between the two devices at controlled environmental conditions. ‘Study 2’ used control materials to assess the concordance between the two devices under varying environmental conditions and also investigated any differences in Ct-values or Tm values for each MTB Ultra probe [6].

Study 1 was carried out on TB clinical (sputum) specimens. FIND provided 160 TB-positive sputum specimens characterized by both phenotypic drug susceptibility testing and whole genome sequencing, of which one was excluded from the study due to insufficient volume for testing on both Omni and GeneXpert (S1 Table). San Raffaele provided 40 negative sputum specimens collected from non-symptomatic individuals at low-risk for TB infection. Informed consent was waived for this study by San Raffaele Scientific Institute Ethical committee, as only remnant, de-identified, decontaminated sputum samples were used, no investigational device test results were provided to the healthcare provider or study participants during the study, and all data were analysed anonymously. One-third (33%; 53/160) of the selected TB-positive specimens were smear-negative. The final TB-positive specimen set contained resistance mutations that captured the majority of observed RIF resistance mechanisms globally (at least 80% of RIF resistance; S2 Table).

For each clinical specimen, aliquots were pooled, homogenized, and then processed according to the MTB Ultra package insert [7]. 2 ml of the sample reagent-treated sample was transferred into two MTB Ultra cartridges which were randomly allocated for testing between GeneXpert and Omni, standing side by side at room temperature conditions (i.e. 20–25°C, 50% relative humidity). Five Omni and two GeneXpert devices were used for Study 1. The concordance between devices was assessed for TB detection and for RIF resistance detection. RIF resistance was defined based upon a composite reference standard: a sample was considered RIF-resistant if phenotypic drug susceptibility testing was resistant and/or a known *rpoB* resistance mutation was identified by whole genome sequencing, whereas a sample was considered RIF-susceptible only if phenotypic drug susceptibility testing was susceptible and no known *rpoB* resistance mutation was identified by whole genome sequencing. Error/invalid rates for each device were also determined.

For Study 2, Cepheid provided aliquots of two MMQCI INTROL^®^ materials (one *rpoB* wildtype strain (‘WT’) and one multidrug-resistant strain with *rpoB* resistance mutations S522L, H526D and S531L (‘MDR2’) at 2e^4^ cells/mL. Equivalence limits for MTB Ultra probe Ct-values were set prior to study initiation using data provided by the manufacturer for five different lots of MTB Ultra cartridges which were tested at 3x the MTB Ultra LoD. For each probe/analyte for each lot, 1.5*standard deviation was determined and the Ct equivalence limit was set at the maximum value seen across the five lots [8]. For Study 2, MTB Ultra cartridges were stored at the respective temperature and humidity conditions for 24 hours prior to testing. For each day of testing, fresh dilutions of MMQCI control stocks were made in TET buffer. Omni testing was conducted for each environmental condition (room temperature and room humidity or 35°C, 90% relative humidity in a Daihan ThermoStable STH temperature humidity chamber) using 30 replicates of the WT control and 30 replicates of the MDR2 control at 3x the MTB Ultra limit of detection (LoD: WT controls were tested at 200cells/mL and MDR2 controls were tested at 400cells/mL). GeneXpert testing was also conducted at room temperature and room humidity using 30 replicates of the WT control and 30 replicates of the MDR2 control at 3x the MTB Ultra LoD, which is the same concentration for which the manufacturer had provided prior testing data for Ultra cartridges, allowing for the assessment of equivalence in this study against set equivalence limits. Daily negative control testing (TET buffer only) was also performed for each device. Two GeneXpert and eight Omni devices were used for Study 2. The concordance between devices was assessed for TB detection and for RIF resistance detection, and the equivalence of Ct values for each probe as well as the difference in Tms for each *rpoB* probe was evaluated. Error/invalid rates for each device were also determined.

## Results

In Study 1, 100% (40/40) of TB-negative specimens and 99.4% (158/159) of TB-positive specimens were accurately characterized by each device (Table 1). One smear-negative, culture-positive specimen was detected on GeneXpert (“MTB Trace DETECTED” result) but not Omni. The sensitivity of MTB Ultra on Omni was thus 0.6% lower than of MTB Ultra on GeneXpert (95%CI -1.7% to +3.5%), with the confidence interval suggesting likely equivalent performance; no discordance in specificity was observed. In addition, 92.9% (145/156) of tested RIF-resistant specimens were accurately characterized by both devices (Table 2). One smear-negative, culture-positive specimen with “very low” TB detection by both devices was characterized as RIF resistance not detected by GeneXpert, despite WGS detection of *rpoB* H445Y (S3 Table). Another specimen that was smear-positive (scanty), culture-positive was characterized as RIF resistance not detected by both GeneXpert and Omni, despite WGS detection of *rpoB* D435G and I491F mutations. Two additional phenotypically RIF-resistant smear-positive, culture-positive specimens with Q432P mutations were mischaracterized as “RIF resistance not detected” by Omni. The sensitivity for RIF-resistance detection (among specimens yielding RIF-calls on both devices) of MTB Ultra on Omni was thus estimated to be 0.7% higher than of MTB Ultra on GeneXpert (95%CI -2.5% to +4.2%) with the confidence interval suggesting likely equivalent performance; specificity was not estimated since only two RIF-sensitive specimens were tested. The proportion of RIF-indeterminate results for Omni and GeneXpert in Study 1 were 3.2% and 3.8%, respectively. For Study 1, the overall error/invalid rate for GeneXpert was 4.5% (10/223; 8 initial errors and 2 initial invalids for four modules of two devices, which resolved upon re-testing), while the error/invalid rate for Omni was 2.7% (6/219; 2 initial errors and 4 invalids for two devices which resolved upon re-testing).

**Table 1:** Overall tuberculosis detection results by Xpert MTB/RIF Ultra on Omni versus GeneXpert devices.

	Result of test	Omni		
		TB+	TB-	Total (%)
**GeneXpert**	**TB+**	158 (79.8%)	1^a^ (0.5%)	**159 (79.9%)**
	**TB-**	0 (0.0%)	40 (20.1%)	**40 (20.1%)**
	**Total (%)**	**158 (79.4%)**	**41 (20.6%)**	**199 (100%)**

TB+: *M*. *tuberculosis* detected, TB-: *M*. *tuberculosis* not detected. All percentages are based on the total number of samples tested between the two platforms (n = 199).

^a^ This discordant specimen was characterized in the FIND specimen bank as smear-negative, culture-positive and was only ‘trace’ positive on MTB Ultra testing on GeneXpert.

**Table 2:** Resistance detection results by Xpert MTB/RIF Ultra on Omni versus GeneXpert devices for rifampicin-resistant specimens.

	Result of test	Omni			
		RIF-R	RIF-S	Indeterminate	Total (%)
**GeneXpert**	**RIF-R**	145 (92.9%)	0 (0.0%)	2 (1.3%)	**147 (93.1%)**
	**RIF-S**	1^a^ (0.6%)	1^b^ (0.6%)	1 (0.6%)	**3 (1.9%)**
	**Indeterminate**	2 (1.3%)	2 (1.3%)	2 (1.3%)	**6 (3.8%)**
	**Total**	**148 (94.9%)**	**3 (1.9%)**	**5 (3.2%)**	**156 (100%)**

RIF-R: rifampicin resistance detected, RIF-S: rifampicin resistance not detected. All percentages are based on the total number of RIF resistant cases for which a RIF resistance call was made by both devices (n = 156).

^a^ This discordant specimen with an *rpoB* H445Y mutation (a known, high-confidence resistance-conferring mutation) was characterized in the FIND specimen bank as phenotypically RIF-resistant, smear-negative, culture-positive and ‘very low’ positive on MTB Ultra testing on both GeneXpert and Omni.

^b^ This discordant specimen with *rpoB* D435G & I491F mutations (known resistance-conferring mutations) was characterized in the FIND specimen bank as phenotypically RIF-resistant, smear-positive (scanty), culture-positive and ‘low’ positive on MTB Ultra testing on both GeneXpert and Omni.

In Study 2, all controls were accurately characterized by each device for all repeats regardless of tested environmental condition. Equivalence of the Cts for all probes was demonstrated based upon prospectively set, pre-defined equivalence limits (S4 and S5 Tables). Within the set equivalence limits, variations were observed, with MTB Ultra having consistently higher Ct and Tm values on Omni than GeneXpert, though these did not compromise the final result output. For Study 2, the overall error/invalid rate for GeneXpert was 4.5% (3/67; 3 initial errors which resolved upon re-testing), while the error/invalid rate for Omni was 1.5% (2/133; 2 initial errors which resolved upon re-testing).

## Discussion

The aim of this study was to evaluate the equivalence of Omni compared to GeneXpert through MTB Ultra testing of well-characterized clinical specimens and controls. The results confirmed high concordance between the Omni and GeneXpert devices in clinical specimens tested at normal environmental conditions as well as for an MMQCI control panel tested at high temperature and humidity conditions, including equivalent Cts for all MTB Ultra probes.

The two devices showed excellent sensitivity and specificity upon MTB Ultra testing of clinical sputum specimens at normal environmental conditions and were highly concordant overall for TB detection, with 99.5% (198/199) of all specimens correctly characterized by both devices. Only one of the more challenging specimens (i.e. 1.9%; 1/53 of the total smear-negative, culture-positive specimens tested) was mischaracterized as TB-negative by Omni. This result is unsurprising considering the low bacterial load in the sputum sample, confirmed by the “trace” call on GeneXpert, and given the uneven distribution of bacteria in sputum specimens. The two devices also demonstrated high overall sensitivity and concordance for RIF resistance detection, with 92.9% (145/156) of all RIF-resistant specimens correctly characterized by both devices. The tested specimens were more often mischaracterized by GeneXpert compared to Omni, though the overall number of mischaracterized specimens was low. There is a possibility that a number of these discordant specimens were heteroresistant, with resistant populations at or below the MTB Ultra threshold for resistance detection, as reference standard-discordances were observed even in MTB Ultra ‘high’ TB-positive specimens and so these calls did not appear to be dependent on bacterial load. The strengths of this assessment included the testing of well-characterized clinical specimens that included both TB-negatives and -positives, with the TB-positives consisting of geographically diverse samples of various smear grades and a variety of RIF resistance mutations, as well as testing between five different Omni devices.

Equivalence of Omni and GeneXpert was also demonstrated for testing of wildtype and mutant controls at both normal and challenging environmental conditions. All TB and RIF resistance calls were correct for each replicate of each control at each environmental condition tested. No differences were observed for testing Omni at normal versus challenging environmental conditions. Cts for each of the probes included in the MTB Ultra assay (i.e. SPC, IS1081-IS6110, rpoB1, rpoB2, rpoB3 and rpoB4) fell within the predetermined equivalency limits. Differences in Ct and Tm values were observed between devices, with Omni consistently having higher Cts and Tms than GeneXpert, but these differences did not compromise TB or RIF resistance detection calls at 3x LoD. The difference between Tms for wildtype vs mutant controls was very large (e.g. on average >4°C for rpoB4), and so the relatively small shifts observed with Omni are unlikely to result in incorrect RIF calls even at the LoD. Ultimately, the Ct differences noted between Omni and GeneXpert are in the range of the variation typically observed between individual modules (whether Omni or GeneXpert) [6], which is reflected in the equivalence limits that were chosen a priori. The strengths of this assessment included the setting of prospective Ct equivalency limits, selection of challenging environmental conditions for testing, and testing of multiple Omni devices to capture a range of performance.

Overall error/invalid rates were <5% for both GeneXpert (4.5%) and Omni (1.5–2.7%) devices. Limitations of this study included the fact that no culture-positive, GeneXpert TB-negative specimens were tested, which would reflect the most challenging specimens for MTB Ultra TB detection. Also, only two RIF-susceptible specimens were tested, as these specimens were not available in the FIND sample bank at the time of this study. However, control testing results demonstrated that the accurate characterization of RIF-susceptible specimens by Omni would likely not be problematic. Additionally, the majority of data for this study were generated using a single GeneXpert device, and so inter-device variability of Cts and Tms was not captured for GeneXpert. Furthermore, it should be noted that data for four runs (1.1% of 354 total Omni runs in this study) failed to be transferred to C360 from two Omni devices while testing controls at challenging environmental conditions, highlighting a potential issue with data transfer that will be important to address during operational research projects.

Field studies comparing the GeneXpert and Omni devices will be essential to corroborate the results of this laboratory validation. Ideally, clinical evaluations should be conducted with representative patient populations, including samples from TB-positive, RIF-susceptible patients which were not well represented in this laboratory study. Field testing in this regard would provide critical data to support specificity, in addition to sensitivity, of Ultra testing on the Omni device. It is also important to acknowledge that Xpert MTB/RIF Ultra testing on Omni will likely have the same noted performance limitations as GeneXpert [9–11], which may have important implications in the field for resistance detection and patient management. In this respect, and especially for peripheral settings, it will be essential to evaluate and report detailed performance findings of all Omni implementation studies, in additional to assessing operational aspects of the Omni platform in settings of intended use.

Overall, in this laboratory validation study, the Omni and GeneXpert devices were highly concordant for MTB Ultra detection of both TB and RIF resistance. Furthermore, the accuracy of MTB Ultra detection of TB and resistance to RIF on Omni was not affected by extreme environmental conditions. These findings support the use of Omni in place of GeneXpert in peripheral settings with infrastructure limitations (i.e. stable power supply) and extreme environmental conditions.

## Supporting information

S1 File Omni bioequivalence study protocol. (PDF)

S2 File (XLSX)

S1 Table Geographical spread and characteristics of tested specimens. (DOCX)

S2 Table Rifampicin resistance-associated mutations in tuberculosis-positive specimens tested in the Omni bioequivalence study. (DOCX)

S3 Table Listing of discordant rifampicin resistance detection results on Omni versus GeneXpert. (DOCX)

S4 Table Equivalence of Xpert Ultra Cts and Tms comparing Omni to GeneXpert at normal environmental conditions. (DOCX)

S5 Table Equivalence of Xpert Ultra Cts and Tms comparing Omni at challenging environmental conditions to GeneXpert at normal environmental conditions. (DOCX)
