# Supplementary material for: Equivalence of the GeneXpert System and GeneXpert Omni System for tuberculosis and rifampicin resistance detection
Source: PLoS One. 2021 Dec 17;16(12):e0261442. doi: 10.1371/journal.pone.0261442 (PMC8682871; doi:10.1371/journal.pone.0261442)
Supplement: S1 File — (PDF) [file pone.0261442.s001.pdf]

CLINICAL TRIAL PROTOCOL

External laboratory bio-equivalency study of the Omni and GeneXpert platforms for TB and RIF resistance detection by Xpert Ultra

Short title

Omni Bio-equivalency Study

Protocol Version Number:

v1.0

Date:

23 July 2020

Disease Programme:

TB

FIND
Because diagnosis matters

FIND
Campus Biotech
Chemin des Mines 9
1202 Geneva, Switzerland
T: +41 (0)22 710 05 90

Confidentiality Statement:

The information contained in this document, especially unpublished data, is the property of FIND (or under its control) and may not be reproduced, published or disclosed to others without prior written authorization from FIND.

Table of Contents

INSTITUTIONS/ORGANIZATIONS/PARTNERS INVOLVED IN THE TRIAL* 5

SIGNATURE PAGE (SPONSOR) 6

STATEMENT OF PRINCIPAL INVESTIGATOR 7

PROTOCOL HISTORY/AMENDMENT SUMMARY* 8

LIST OF ABBREVIATIONS AND ACRONYMS 9

PROTOCOL SYNOPSIS 11

SCHEDULE OF ACTIVITIES 13

1 INTRODUCTION 14

1.1 TRIAL RATIONALE 14
1.2 BACKGROUND 14
1.3 BENEFIT/RISK ASSESSMENT 15

2 STUDY OBJECTIVES AND ENDPOINTS 15

3 STUDY DESIGN 16

3.1 GENERAL DESIGN 16
3.2 SCIENTIFIC RATIONALE FOR TRIAL DESIGN 16

4 STRAIN SELECTION AND ELIGIBILITY 16

5 INVESTIGATIONAL PRODUCTS 17

5.1 INVESTIGATIONAL PRODUCT 17
5.2 PREPARATION/HANDLING/STORAGE/ACCOUNTABILITY 17
5.3 MINIMISATION OF ERROR AND BIAS 18

6 STUDY PROCEDURES 19

6.1 STUDY WORKFLOW 19
6.2 PANEL AND STRAIN, HANDLING, TRANSPORT AND STORAGE 20
6.3 COMPARATOR TEST AND INDEX TEST PROCEDURES 20
6.4 SAFETY ASSESSMENTS 21

7 SAFETY AND INCIDENT REPORTING 21

8 STATISTICAL CONSIDERATIONS 21

8.1 STATISTICAL ANALYSIS PLAN 21
8.2 SAMPLE SIZE DETERMINATION 23

9 REGULATORY AND ETHICAL CONSIDERATIONS 25

9.1 REGULATORY AND ETHICS APPROVALS 25
9.2 FINANCIAL DISCLOSURE 25

10 DATA HANDLING AND RECORD KEEPING 26

10.1 SOURCE DATA AND SOURCE DOCUMENTS 26
10.2 DATA MANAGEMENT 26

11 QUALITY MANAGEMENT 27

11.1 QUALITY CONTROL (MONITORING) 27
11.2 TRIAL AND SITE CLOSURE 27

12 PUBLICATION POLICY 28

13 REFERENCES 29

14 APPENDICES 30

APPENDIX 1: PROTOCOL AMENDMENT SUMMARY TABLE 31
APPENDIX 2: PACKAGE INSERT FOR MMQCI CONTROLS 32

Institutions/Organizations/Partners Involved in the Trial*

The main institutions/organizations/partners are listed below. More information can be found in the Study Contact List for the names, telephone, e-mail address, title and role on the study.

Organization/Institution/Company/Partner	*Role in the Trial*
FIND, Geneva, Switzerland	Sponsor
San Raffaele Scientific Institute, Milan, Italy	Laboratory
Cepheid, Sunnyvale, CA, USA	IVD Manufacturer

FIND (Sponsor)

Contact person: Samuel Schumacher

Campus Biotech

Chemin des Mines 9

1202 Geneva

Switzerland

San Raffaele Scientific Institute

Principal Investigator: Daniela Cirillo

Via Olgettina 58

20132 Milano

Italy

Cepheid

Contact person: Vish Kulkarni

1324 Chesapeake Terrace

Sunnyvale, CA 94089

U.S.A.

*Terms of references and nature of agreements are available from FIND on request.

Signature Page (Sponsor)

We, the undersigned, have developed, reviewed and approved this protocol, including appendices. We will supervise and coordinate the clinical trial according to the principles outlined in the Declaration of Helsinki and Good Clinical Practice and in compliance with applicable regulatory requirements.

HEAD OF TB PROGRAMME

Name: Morten Ruhwald

Signature: ______________________ Date: 24/07/2020

DD/MMM/YYYY

TRIAL MANAGER

Name: Sophia Georghiou

Signature: ______________________ Date: 23/07/2020

DD/MMM/YYYY

PROJECT MANAGER

Name: Samuel Schumacher

Signature: ______________________ Date: 24/07/2020

DD/MMM/YYYY

Statement of Principal Investigator

In signing this page, I, the undersigned, agree to conduct the trial according to the protocol and ICH-GCP E6 (R2) guidelines and in compliance with applicable regulations.

I will ensure that the requirements relating to obtaining Institutional Review Board (IRB)/ Independent Ethics Committee (IEC) review and approval are met. I will promptly report to the IRB/IEC any and all changes in the research activities covered by this protocol.

I have sufficient time to properly conduct and complete the trial within the agreed trial period and I have adequate resources (staff and facilities) for the foreseen duration of the trial.

I am responsible for supervising any individual or party to whom I delegate trial related duties and functions conducted at the trial site. Further, I will ensure this individual or party is qualified to perform those trial-related duties and functions.

I certify that key individuals involved with the conduct of this trial, including myself, have completed GCP training and, if applicable, Human Subjects Protection Training.

I understand that all information obtained during the conduct of the trial with regard to the subjects' state of health will be regarded as confidential. No participant's names or personal identifying information may be disclosed. All participant data will be anonymized and identified by assigned numbers on all Case Report Forms, laboratory samples and other trial related information (such as essential documents) forwarded to FIND. Monitoring and auditing by FIND, and inspection by the appropriate regulatory authority(ies), will be permitted.

I will maintain confidentiality of this protocol and all other related investigational materials. Information taken from the trial protocol may not be disseminated or discussed with a third party without the express consent of FIND.

Name of Principal Investigator: ______________________________
(Print)

Name:

Signature: ______________________________ Date: ______________________

DD/MMM/YYYY

Protocol History/Amendment Summary*

Version number	Release date	Comments
0.1		Initial draft version
0.2		Added dilution of controls to close to LoD in second sub-assessment
0.3		Removed dilution of controls at LoD- focus analysis on Cts and Tms
1.0		Finalized after sharing with WHO

*Refer to Appendix 1 for Protocol Amendment History

List of Abbreviations and Acronyms

**Abbreviation/acronym**	**Meaning**
AE	Adverse Event
CRF	Case Report Form
GCP	Good Clinical Practice
GCLP	Good Clinical Laboratory Practice
GDoP	Good Documentation Practice
ICF	Informed Consent Form
IDMC	Independent Data-Monitoring Committee
IEC	Independent Ethics Committee
ICH	International Council on Harmonisation
IRB	Institutional Review Board
ISF	Investigator Site File
ISO	International Organisation for Standardization
IVD	In Vitro Diagnostic
MDR	Multidrug-resistant
MTB	*Mycobacterium tuberculosis*
MTBC	*Mycobacterium tuberculosis* complex
QA	Quality Assurance
QC	Quality Control
QMS	Quality Management System
RA	Regulatory Authority
RIF	Rifampicin
RBM	Risk Based Monitoring
RM	Risk Management
SAP	Statistical Analysis Plan
SAE	Serious Adverse Event
SOA	Schedule of Activities

SOP	Standard Operating Procedure
TB	Tuberculosis
TMF	Trial Master File
WHO	World Health Organisation
XDR	Extensively drug-resistant

Protocol Synopsis

**Title**	**External laboratory bio-equivalency study of the Omni and GeneXpert platforms for TB and RIF resistance detection by Xpert Ultra**
**Short title**	Omni Bio-equivalency Study
**Protocol version and date**	V1.0
**Background and rationale**	The placement of GeneXpert in peripheral settings is limited by the lack of sufficient infrastructure (i.e. stable power supply) and by variable environmental conditions (i.e. temperature and humidity fluctuations). GeneXpert Omni is a new robust platform for Xpert cartridge testing at primary healthcare facilities. The Omni device is a single module, battery-powered, point-of-care device with cloud-based connectivity for data transfer and increased stability to dust and high temperatures. Evaluation of the Omni device against the well-characterized GeneXpert platform is needed to determine the equivalency of Omni for the detection of *M. tuberculosis* (MTB) and resistance to rifampicin (RIF). In particular, evaluation of the Omni device under environmental stressors (i.e. high temperature and humidity) is needed to demonstrate whether the device can be used at point-of-care with equivalent performance to GeneXpert.
**Primary objective(s)**	1.1 Compare the performance of the Xpert MTB/RIF Ultra assay between the GeneXpert and Omni systems with testing of clinical specimens<br>1.2 Compare the performance of the Xpert MTB/RIF Ultra assay between the GeneXpert and Omni systems with testing of MMQCI controls at high temperature and humidity conditions
**Primary endpoints (outcomes)**	1.1 Positive and negative concordance of Xpert MTB/RIF Ultra testing on Omni compared to GeneXpert for clinical specimens<br>1.2 Positive and negative concordance of Xpert MTB/RIF Ultra testing on Omni compared to GeneXpert for MMQCI controls at different temperature and humidity conditions<br>1.3 Average difference in Ct-values for each Xpert MTB/RIF Ultra MTBC probe when testing on Omni compared to GeneXpert for MMQCI controls at different temperature and humidity conditions<br>1.4 Average difference in Tm values for each Xpert MTB/RIF Ultra *rpoB* probe when testing on Omni compared to GeneXpert for MMQCI controls at different temperature and humidity conditions
**Study design**	This is an external laboratory equivalency study to evaluate the performance Xpert MTB/RIF Ultra testing on Omni compared to GeneXpert. This study will use well-characterized clinical specimens from existing sample banks to assess the positive and negative concordance between the two devices at normal environmental conditions.<br>The study will also evaluate the positive and negative concordance between the two devices at high heat and humidity using Cepheid controls, and look at differences in Tm and Ct values between platforms for Ultra testing of the same controls at 3x Ultra LoD.

**Study sites/setting**	San Raffaele Scientific Institute, Milan, Italy
**Study population**	Clinical specimens selected from the FIND specimen bank and provided MMQCI controls
**Sample Size**	Objective 1.1: Xpert MTB/RIF Ultra testing of a total of 160 MTB positive and 40 MTB negative specimens on both Omni and GeneXpert<br>Objective 1.2: Xpert MTB/RIF Ultra testing of a total of 30 replicates of the MTB WT control and 30 replicates of the MTB MDR control at 3x the Ultra LoD per each environmental condition on Omni and at baseline conditions on GeneXpert<br>One negative control will also be tested per day per Omni/Gene Xpert device.
**Study duration**	5 months from conception to completion, 30 days of testing.
**Time schedule**	Study set up (IRB approval, study agreements) 8 weeks<br>Shipment of devices, samples, Cepheid controls and cartridges 3 weeks<br>Testing and data reporting 6 weeks<br><br>Start Q2 2020

Schedule of Activities

Procedure	Timeline	Notes
Study set up (IRB approval, study agreements)	1 December – 15 July 2020	*Cepheid Omni delay has impacted timelines. No instruments available before July
Shipment of devices, samples, Cepheid controls and cartridges	16 July – 6 August 2020	*Additional time allocated for shipments given COVID-related delays
Testing and data reporting	7 August – 18 Sept 2020	

1 Introduction

The GeneXpert Omni system (‘Omni’) is a single-cartridge, battery-powered, point-of-care platform for rapid, onsite molecular tuberculosis testing at primary care clinics in high burden countries.

The study described herein is an external, laboratory study to investigate the equivalency of Xpert MTB/RIF Ultra testing on the Omni platform compared to the GeneXpert platform.

1.1 Trial Rationale

A well-controlled laboratory evaluation study is needed to evaluate the performance of Xpert MTB/RIF Ultra testing on the Omni system compared to the GeneXpert platform to ultimately determine platform concordance for the detection of *M. tuberculosis* (MTB) and resistance to rifampicin (RIF). An evaluation of Xpert MTB/RIF Ultra testing on the Omni device under high temperature and humidity conditions is also needed to support the use of the platform at the point-of-care in settings of intended use.

1.2 Background

There are 10 million incident cases of tuberculosis (TB) annually, though it is estimated that 3 million of these cases go undiagnosed each year[1]. Additionally, the emergence of multi- and extensively drug-resistant TB (M/XDR-TB) is a major threat to global TB control. Standard phenotypic methods for TB diagnosis are inadequate, as culture and conventional drug susceptibility testing (DST) using media such as Lowenstein–Jensen and Middlebrook 7H10/11 can take up to 8-12 weeks to return results[2], and even liquid-based culture techniques such as MGIT® (BD Diagnostics) still take several weeks[3]. The delays associated with culture-based DST lead to prolonged periods of ineffective TB therapy and ongoing transmission. In view of the inadequate diagnosis and increasing incidence of M/XDR-TB, the development of rapid molecular diagnostic tests for the identification of MTB and the detection of drug resistance has become a research and implementation priority.

The Xpert® MTB/RIF Assay (‘Xpert’) provides a result for TB and RIF resistance detection in a sputum sample within 2 hours with minimal hands-on time[4,5]. Xpert sensitivity for TB detection is 98% for smear-positive and 67% for smear-negative TB, with a specificity of 99% based upon 27 studies with close to 10,000 participants. The assay detects RIF resistance with 95% sensitivity and 98% specificity[6]. In 2010, WHO endorsed Xpert for use as the initial diagnostic test in individuals suspected of MDR or HIV-associated TB, and in 2014 expanded this recommendation for use in all patients, given adequate resources[7]. The Xpert MTB/RIF Ultra assay, incorporating two different multicopy amplification targets (IS6110 and IS1081) and using a renewed assay chemistry coupled with a larger DNA reaction tube, has improved upon the Xpert MTB/RIF assay, demonstrating enhanced accuracy for RIF resistance detection and greatly improved sensitivity compared with that of Xpert in smear-negative, culture-positive TB and HIV-associated TB

meningitis[8,9]. These assays have since been widely used in TB programmes, but diagnostic gaps remain, especially at lower-level healthcare facilities.

The placement of GeneXpert in peripheral settings is limited by a lack of sufficient infrastructure (i.e. stable power supply) and by extreme environmental conditions[10]. The GeneXpert Omni is a new, robust platform to allow Xpert MTB/RIF or Ultra testing at primary healthcare facilities. The Omni device is a single-module, battery powered, point-of-care device with cloud-based connectivity for data transfer. The device has increased stability to dust and high temperatures. To date, alpha prototype units of the Omni system have undergone preliminary assessments of the user interface and connectivity features in Malawi and South Africa, and verification and validation of the Omni instrument and its associated software (including its performance against GeneXpert) will be performed by the manufacturer. An external, laboratory study conducted independently of the manufacturer is also needed to assess the equivalency of the Omni platform compared to GeneXpert.

1.3 Benefit/Risk Assessment

No participants will be recruited during this study and so no risks are expected. In contrast, the knowledge gained from this study will potentially benefit society by improving TB and drug resistance detection.

2 Study Objectives and Endpoints

The aim of the study is to assess whether Xpert MTB/RIF Ultra testing on the Omni platform is equivalent to Xpert MTB/RIF Ultra testing on the GeneXpert platform.

Objectives	Outcomes
Primary	
1.1 Compare the performance of the Xpert MTB/RIF Ultra assay between the GeneXpert and Omni systems with testing of clinical specimens<br>1.2 Compare the performance of the Xpert MTB/RIF Ultra assay between the GeneXpert and Omni systems with testing of MMQCI controls at high temperature and humidity conditions	1.1 Positive and negative concordance of Xpert MTB/RIF Ultra testing on Omni compared to GeneXpert for clinical specimens<br>1.2 Positive and negative concordance of Xpert MTB/RIF Ultra testing on Omni compared to GeneXpert for MMQCI controls at different temperature and humidity conditions<br>1.3 Average difference in Ct-values for each Xpert MTB/RIF Ultra MTBC probe when testing on Omni compared to GeneXpert for MMQCI controls at different temperature and humidity conditions<br>1.4 Average difference in Tm values for each Xpert MTB/RIF Ultra rpoB probe when testing on Omni compared to GeneXpert for MMQCI controls at different temperature and humidity conditions

3 Study Design

3.1 General Design

This is an external, laboratory-based equivalency study of the Cepheid GeneXpert and Omni platforms. This study will use banked clinical specimens to assess the positive and negative concordance between the two devices. The study will also evaluate the Omni device performance upon testing of Xpert MTB/RIF Ultra cartridges in comparison to GeneXpert for high temperature and humidity conditions using MMQCI controls, assessing the positive and negative concordance between the two devices at 3x the Ultra LoD given different environmental and determining the average difference in Ct-values or Tm values for each Xpert MTB/RIF Ultra probe, as appropriate.

3.2 Scientific Rationale for Trial Design

This external, well-controlled laboratory assessment study will provide equivalency data for testing of Xpert MTB/RIF Ultra on the Omni platform compared to GeneXpert under controlled environmental conditions as well as high temperature and humidity conditions. The evaluation will be carried out independently of the manufacturer at San Raffaele Scientific Institute, Milan, Italy.

4 Strain selection and Eligibility

For the equivalency assessment that is carried out at controlled environmental conditions, FIND will provide the site with a set of clinical specimens from the FIND specimen bank. The selection will include 160 MTB culture-positive raw sputum specimens. Forty MTB culture-negative sputum specimens will also be provided by San Raffaele for this assessment. The rationale for using raw clinical specimens is that (a) the specimens are well characterized and (b) it allows for end-use testing of the platforms (i.e. testing of clinical sputum samples).

For the equivalency assessment that is carried out at high environmental conditions, Cepheid will provide the site with aliquots of controls (Appendix 2: MTBC-positive wildtype, MTBC-positive MDR and MTBC-negative). Each environmental condition will be tested by 30 replicates of the wildtype control and 30 replicates of the MDR control at the Ultra LoD. Daily negative control testing will be performed per GeneXpert and Omni device. The rationale for using Cepheid controls instead of clinical specimens for this assessment is that (a) high environmental (i.e. temperature and humidity) conditions, rather than the DNA extraction process itself, are a challenge for the assay and (b) controls will introduce less variability as the bacillary load can be controlled.

5 Investigational products

5.1 Investigational Product

The investigational product used in this study is the GeneXpert Omni platform. The Xpert MTB/RIF Ultra assay will be tested on this platform for the detection of *M. tuberculosis* and resistance to RIF. The comparator product used in this study is the GeneXpert platform.

Instructions for use of the investigational product(s) are provided by the manufacturer.

Medical device incidents, including those resulting from malfunctions of the device or IVD assay must be detected, documented and reported by the investigator at each site throughout the trial (see Study Manual).

5.2 Preparation/Handling/Storage/Accountability

The investigational products will be strictly accounted for, including receipt and inventory, storage, use during the trial, and return or disposal, as detailed in the Manual of Procedures.

Acquisition

Procurement of the investigational products will be done through FIND, who will coordinate shipments from the manufacturer. It is the responsibility of the study site to maintain an updated inventory of the study materials and to inform FIND immediately if additional materials are required.

The investigator or designee must confirm appropriate temperature conditions have been maintained during transit for the investigational product received and any discrepancies are reported and resolved before its use.

Storage

Procedures for product storage and disposal will be described in the Study Manual.

The investigational product must be stored in a secure, environmentally controlled, and monitored (manual or automated) area in accordance with the labelled storage conditions with access limited to the investigator and authorized site staff. Expired or unused investigational materials will either be discarded or returned to the manufacturer, per manufacturer instructions.

Test Handling and Performance

Testing using the investigational products will be performed according to the manufacturer's instructions and as further outlined within the Study Manual.

Accountability

The investigator is responsible for trial intervention accountability, reconciliation, and record maintenance (i.e., receipt, reconciliation, and final disposition records). Investigational Product Accountability logs filled at each site will ensure the proper follow-up of the used, failed and remaining investigational products

Further guidance and information for the final disposition of unused investigational product are provided in the Study Manual.

Export and Import Permits

It is expected that most countries will require import permits for receiving the investigational materials. Local sites are responsible for making import permit applications in a timely manner.

Quality Control Check for Incoming Shipments

Upon arrival of each new shipment of assays, the sites will conduct and document an incoming quality check following the Study Manual. New lots may only be used after this quality check is successfully passed.

Local procurement

The investigational site is responsible for assessing its needs and procuring any supplies, reagents and kits needed for the study that are locally available in order to include these costs in the study budget, other than the supplies of the assays under investigation, which will be supplied free of charge by the manufacturer.

It is expected that the investigational site will require import permits for receiving the investigational products. The site is responsible for making import permit applications in a timely manner.

It is the responsibility of sites to conduct and document incoming quality check for each new shipment upon arrival and following the study manual. Incoming quality check includes visual inspection of the materials and a check that materials were received at conditions specified by the manufacturer as well as successful testing of external controls. New lots and new shipments may only be used after this quality check is successfully passed.

5.3 Minimisation of Error and Bias

For the equivalency assessment of Omni against GeneXpert at controlled environmental conditions, each sputum specimen will be prepared and split into two aliquots, then randomized (see section 6: Study procedures) and loaded into two Xpert MTB/RIF Ultra cartridges. One cartridge will be tested using GeneXpert and one using Omni. In this way, both devices will be tested with the same sample and there is no risk that any of the two devices will be tested on a higher-quality or more concentrated sample.

Cepheid controls will be used for the equivalency assessment of the Omni at high environmental conditions (i.e. high temperature and humidity). There is therefore minimal risk that any of the two devices will be tested on a higher-quality or more concentrated sample.

Compiling molecular test results harbours little risk of review bias, as results output from Xpert MTB/RIF Ultra testing is automatic, not requiring manual reading or user interpretation.

6 Study Procedures

Trial procedures and their timing are summarized in the Schedule of Assessments. Protocol waivers or exemptions are not allowed.

Adherence to the trial design requirements, including those specified in the Schedule of Assessments, is essential and required for study conduct.

6.1 Study workflow

The study flow is shown in Figure 1.

FIND will ship the required clinical specimens selected for this study to the investigational site. Cepheid will ship the platforms, cartridges, and MMQCI controls required for this study to the site.

For the equivalency assessment of Omni against GeneXpert at controlled environmental conditions, each clinical specimen will be prepared, homogenized and split into two equal aliquots for Xpert MTB/RIF Ultra testing. The two Xpert MTB/RIF Ultra cartridges will be randomized for testing, with one cartridge tested using the Omni platform and one cartridge tested using the GeneXpert platform at room temperature conditions.

For the equivalency assessment that is carried out at high environmental conditions, Xpert MTB/RIF Ultra cartridges will be stored at the respective temperature and humidity conditions for 72 hours prior to testing. Each environmental condition will be tested by Omni using 30 replicates of the wild type control and 30 replicates of the MDR control at 3x the Ultra LoD. GeneXpert testing using Xpert MTB/RIF Ultra will also be performed using 30 replicates of the wild type control and 30 replicates of the MDR control at 3x the Ultra LoD. Four Omni systems will be used for testing at each environmental condition. Daily negative control testing will also be performed for each device.

Figure 1: Sample workflow

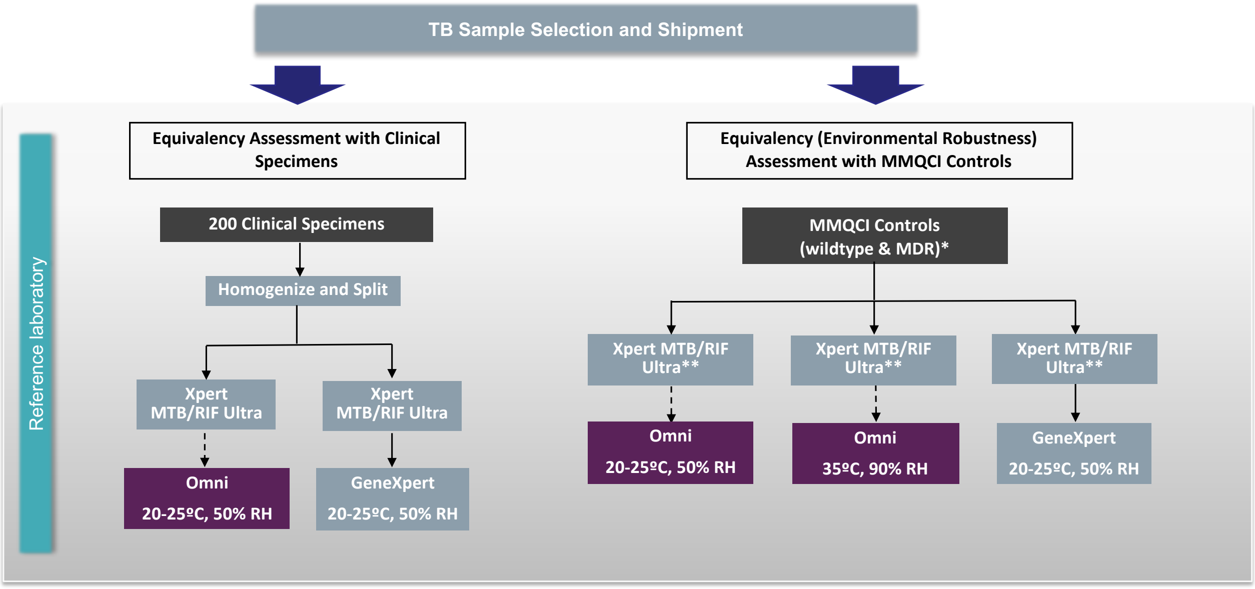

*30 replicates of each control will be tested by each platform at the listed conditions (i.e. 30 replicates per condition) at 3x LoD. Replicate testing will be divided between 4 Omni instruments. Daily negative control testing will also be performed for Omni and GeneXpert.
**Ultra cartridges to be stored at respective condition (20-25C, 50% RH or 35C, 90% RH) for 72hr prior to testing

6.2 Panel and strain, Handling, Transport and Storage

The raw clinical specimens contain live and pathogenic *M. tuberculosis* strains that were collected from tuberculosis patients. Processing of the clinical specimens requires BSL-3 facility. Frozen aliquots of clinical specimens must be stored at -80°C.

Cepheid-provided MMQCI controls (Appendix 2) should be stored at 2-8°C upon receipt and after opening. These controls should not be frozen. Unopened controls are stable through the expiration date printed on each bottle when stored at 2° – 8°C. Opened material tightly capped and returned to the refrigerator (2° – 8°C) shortly after use is stable for thirty (30) days from the date of opening.

6.3 Comparator Test and Index Test Procedures

Xpert MTB/RIF Ultra testing on the GeneXpert and Omni platforms will be performed based on the instructions provided by the manufacturers. Additional work instructions will be prepared by FIND to standardise any areas open to interpretation or variability including sample handling and labelling, sample randomization, quality assurance, training, recording and reporting, and instrument verification.

6.4 Safety Assessments

No safety assessment will be done since no incidents are expected upon use of the different assays (see section 7).

7 Safety and Incident Reporting

Safety and/or incident reporting is not applicable for this study. No participants will be enrolled during this study and the investigational products are non-invasive molecular assays. Therefore, no safety and/or incident reporting is expected during this study.

8 Statistical Considerations

8.1 Statistical Analysis Plan

Given the study design, no detailed statistical analysis plan (SAP) will be prepared for this work. Rather, dummy tables for results reporting will be provided and only some key elements are presented here. This section is a summary of the planned statistical analyses of the primary and secondary endpoints.

Study	Primary Endpoint	Statistical Analysis methods
1	1.1 Positive and negative concordance of Omni compared to GeneXpert when Xpert MTB/RIF Ultra testing is done using clinical specimens	Number of MTB culture positive specimens detected as MTB positive by Xpert MTB/RIF Ultra when using Omni and GeneXpert, respectively<br>Number of MTB culture negative specimens detected as MTB negative by Xpert MTB/RIF Ultra when using Omni and GeneXpert, respectively
2	1.2 Positive and negative concordance of Omni compared to GeneXpert when Omni Xpert MTB/RIF testing is done at high environmental conditions	Number of MTB positive controls detected as MTB positive by Xpert MTB/RIF Ultra when testing Omni at different environmental conditions<br>Number of MTB positive controls detected as MTB positive by Xpert MTB/RIF Ultra when testing Omni vs. GeneXpert at normal environmental conditions<br>Number of RIF-resistant controls detected as RIF resistant by Xpert MTB/RIF Ultra when testing Omni at different environmental conditions<br>Number of RIF-sensitive controls detected as RIF sensitive by Xpert MTB/RIF Ultra when testing Omni at different environmental conditions

		Number of RIF-resistant controls detected as RIF resistant by Xpert MTB/RIF Ultra when testing Omni vs. GeneXpert at normal environmental conditions<br><br>Number of RIF sensitive controls detected as RIF sensitive by Xpert MTB/RIF Ultra when testing Omni vs. GeneXpert at normal environmental conditions
	1.3 Average difference in Ct-values for each Xpert MTB/RIF Ultra MTBC probe when testing on Omni compared to GeneXpert for MMQCI controls at different temperature and humidity conditions	Individual value plots of all Cts from each probe (SPC, IS1081-IS6110, rpoB1, rpoB2, rpoB3, rpoB4) from wildtype MMQCI control testing between GeneXpert and Omni when testing at normal environmental conditions.<br><br>Individual value plots of all Cts from each probe (SPC, IS1081-IS6110, rpoB1, rpoB2, rpoB3, rpoB4) from mutant MMQCI control testing between GeneXpert and Omni when testing at normal environmental conditions.<br><br>Individual value plots of all Cts from each probe (SPC, IS1081-IS6110, rpoB1, rpoB2, rpoB3, rpoB4) from wildtype MMQCI control testing between GeneXpert and Omni when testing at different environmental conditions.<br><br>Individual value plots of all Cts from each probe (SPC, IS1081-IS6110, rpoB1, rpoB2, rpoB3, rpoB4) from mutant MMQCI control testing between GeneXpert and Omni when testing at different environmental conditions.
	1.4 Average difference in Tm values for each Xpert MTB/RIF Ultra rpoB probe when testing on Omni compared to GeneXpert for MMQCI controls at different temperature and humidity conditions	Individual value plots of all Tms from each probe (SPC, IS1081-IS6110, rpoB1, rpoB2, rpoB3, rpoB4) from wildtype MMQCI control testing between GeneXpert and Omni when testing at normal environmental conditions.<br><br>Individual value plots of all Tms from each probe (SPC, IS1081-IS6110, rpoB1, rpoB2, rpoB3, rpoB4) from mutant MMQCI control testing between GeneXpert and Omni when testing at normal environmental conditions.<br><br>Individual value plots of all Tms from each probe (SPC, IS1081-IS6110, rpoB1, rpoB2, rpoB3, rpoB4) from wildtype MMQCI control testing between GeneXpert and Omni when testing at different environmental conditions.<br><br>Individual value plots of all Tms from each probe (SPC, IS1081-IS6110, rpoB1, rpoB2, rpoB3, rpoB4) from mutant MMQCI control testing between GeneXpert and Omni when testing at different environmental conditions.

8.2 Sample Size Determination

Objective 1.1 – Compare the performance of the Xpert MTB/RIF Ultra assay between the GeneXpert and Omni systems with testing of clinical specimens

Table 1. Sample size required to assess percent agreement between platforms at a given confidence interval width with 95% confidence.

% Agreement between platforms	Width of Confidence Interval						
	**5%**	**7.5%**	**10%**	**12.5%**	**15%**	**17.5%**	**20%**
**99%**	61	28	16	10	7	5	4
**97.5%**	150	67	38	24	17	13	10
**95%**	292	130	73	47	33	24	19
**92.5%**	427	190	107	69	48	35	27
**90%**	554	246	139	89	62	46	35
**87.5%**	673	299	169	108	75	55	43
**85%**	784	349	196	126	88	64	49
**82.5%**	888	395	222	142	99	73	56
**80%**	984	438	246	158	110	81	62
**77.5%**	1072	477	268	172	120	88	67
**75%**	1153	513	289	185	129	95	73
**72.5%**	1226	545	307	197	137	101	77
**70%**	1291	574	323	207	144	106	81
**67.5%**	1349	600	338	216	150	111	85
**65%**	1399	622	350	224	156	115	88
**62.5%**	1441	641	361	231	161	118	91
**60%**	1476	656	369	237	164	121	93
**57.5%**	1503	668	376	241	167	123	94
**55%**	1522	677	381	244	170	125	96
**52.5%**	1533	682	384	246	171	126	96

	50%	1537	683	385	246	171	126	97

The GeneXpert and Omni platforms are anticipated to demonstrate equivalent performance for the testing for Xpert Ultra cartridges. Given a confidence level of 95%, a sample size of 150 would yield a confidence interval of 5% to determine 97.5% agreement between platforms. Testing 160 MTB positive sample size would ensure these parameters are met even given a 6.25% error/invalid rate.

Objective 1.2 – Compare the performance of the Xpert MTB/RIF Ultra assay between the GeneXpert and Omni systems with testing of MMQCI controls at high temperature and humidity conditions

Table 2. Sample size required to assess percent agreement between platforms at a given confidence interval width with 90% confidence.

		Width of Confidence Interval						
		**5%**	**7.5%**	**10%**	**12.5%**	**15%**	**17.5%**	**20%**
% Agreement between platforms	**99%**	43	20	11	7	5	4	3
	**97.5%**	106	47	27	17	12	9	7
	**95%**	206	92	52	33	23	17	13
	**92.5%**	301	134	76	49	34	25	19
	**90%**	390	174	98	63	44	32	25
	**87.5%**	474	211	119	76	53	39	30
	**85%**	552	246	138	89	62	46	35
	**82.5%**	625	278	157	100	70	52	40
	**80%**	693	308	174	111	77	57	44
	**77.5%**	755	336	189	121	84	62	48
	**75%**	812	361	203	130	91	67	51
	**72.5%**	864	384	216	139	96	71	54
	**70%**	910	405	228	146	102	75	57
	**67.5%**	950	423	238	152	106	78	60
	**65%**	985	438	247	158	110	81	62

	**62.5%**	1015	451	254	163	113	83	64
	**60%**	1039	462	260	167	116	85	65
	**57.5%**	1058	471	265	170	118	87	67
	**55%**	1072	477	268	172	120	88	67
	**52.5%**	1080	480	270	173	120	89	68
	**50%**	1083	481	271	174	121	89	68

Given a confidence level of 90%, a sample size of 120 would yield a confidence interval of 10% to determine 87.5% agreement between platforms, or a confidence interval of <5% to determine 97.5% agreement between platforms.

9 Regulatory and Ethical Considerations

9.1 Regulatory and Ethics Approvals

No human subjects will be enrolled for this study, and so no ethical issues are expected. The specimens used for this study were obtained from tuberculosis patients who authorized use of their material. To ensure confidentiality, the patient names will not be disclosed in any document.

Any amendments to the protocol must be approved prior to their implementation. The study will not start before the signature of the clinical study protocol of each contractual party involved has been obtained.

Clinical specimens and strains will be shipped to sites in adherence to international transport regulations and safety standards. Handling and manipulation of M. tuberculosis strains will be performed in an adequate biosafety laboratory (BSL-3) to minimize risk of contamination for the technical personnel and the environment. Given the nature of the study, termination due to safety or other reasons is not anticipated. In parallel, knowledge gained from this study may benefit society by improving TB and drug resistance diagnosis.

9.2 Financial Disclosure

Investigators and sub-investigators will provide FIND with sufficient, accurate financial information as requested to allow FIND to submit complete and accurate financial certification or disclosure statements to the appropriate regulatory authorities.

10 Data Handling and Record Keeping

FIND is responsible for the data management of this trial including quality control checks of the data and assessment of overall protocol compliance. Data will be exported from the Omni and Gene Xpert instruments and converted by FIND into a format applicable to the statistical software that will be used for the statistical analysis.

The investigator must permit study-related monitoring, audits, IRB/IEC review, and regulatory agency inspections and provide direct access to source data documents.

FIND or designee is responsible for the data management of this study including quality checking of the data.

Records and documents, pertaining to the conduct of this study must be retained by the investigator for at least 5 years after study completion unless local regulations or institutional policies require a longer retention period. No records may be destroyed during the retention period without the written approval of FIND. No records may be transferred to another location or party without written notification to FIND.

10.1 Source Data and Source Documents

Source documents provide evidence for the existence of the data and substantiate the integrity of the data collected. Source documents are filed at the investigator's site. For this study the source documents will be the results generated by the Omni and Gene Xpert instruments.

10.2 Data Management

Data Management procedures at FIND, including the setup of the database, programming edit and range checks and querying, are described in the Data Management Plan.

Test results from the Omni and Gene Xpert devices will be exported from the investigational device and electronic files will be transferred to FIND for further analysis. FIND will setup a secure data transfer system such as a File Transfer Protocol (FTP) server. Details will be provided in the Trial Manual.

No information concerning the trial or the data generated from the trial will be released to any unauthorized third party without prior written approval of FIND.

The study monitor or other authorized representatives of FIND or regulatory agencies may inspect all documents and records required to be maintained by the co-investigators.

Additional details relating to data entry procedures and timelines will be provided to the investigational site in the Data Management Plan.

11 Quality Management

Quality Management for this study consists of Quality Control activities, training and capacity building provided by FIND (or designee) to the investigational sites and laboratories, as well as the use of Standard Operating Procedures, Work Instructions, Tools and Templates.

Training on the protocol, will be provided by FIND and training on the different assays will be provided by the manufacturers, if this step has not been part of an earlier program. A Study Manual which describes all of the sample testing procedures will be provided by FIND prior to the commencement of the study.

Any missing data or data anomalies will be communicated to the site for clarification and resolution. The site will provide direct access to all study-related records, source data/documents, and reports for the purpose of monitoring and auditing by FIND and inspection by local and regulatory authorities, if requested.

11.1 Quality Control (monitoring)

Quality control should be applied to each stage of data handling to ensure that all data are reliable and have been processed correctly. The investigational site is responsible for performing regular Quality Control checks on the data they generate.

FIND will perform risk-based monitoring of this trial, and associated Quality Control checks, as described in the Monitoring Plan. Quality Assurance (auditing)

As part of routine Quality Assurance, FIND or designee may conduct an audit of the investigational site.

11.2 Trial and Site Closure

FIND designee reserves the right to close the study site or terminate the study at any time for any reason at the sole discretion of FIND. Study sites will be closed upon study completion. A study site is considered closed when all required documents and study supplies have been collected and a study-site closure visit has been performed.

The investigator may initiate study-site closure at any time, provided there is reasonable cause and sufficient notice is given in advance of the intended termination.

Reasons for the early closure of a study site by FIND or investigator may include but are not limited to:

- Failure of the investigator to comply with the protocol or local health authorities, FIND's procedures, including the failure to meet capacity requirements outlined at the start of the study.
- Discontinuation of further study intervention development

12 Publication Policy

Authorship for scientific publication of the trial results will be determined by mutual agreement and in line with International Committee of Medical Journal Editors authorship requirements, as described in the publication policy section of the contractual agreement.

Study results will be presented at international scientific conferences, published in international peer-reviewed journals and presented to WHO for review. Reporting will be in accordance to STARD guidelines.

13 References

1. WHO. *Global TB Report 2019*.; 2019.

2. Heifets L, Cangelosi GA. Drug susceptibility testing of Mycobacterium tuberculosis: A neglected problem at the turn of the century. *Int J Tuberc Lung Dis*. 1999.

3. Small PM, Pai M. Tuberculosis diagnosis - Time for a game change. *N Engl J Med*. 2010. doi:10.1056/NEJMe1008496

4. Boehme CC, Nabeta P, Hillemann D, et al. Rapid molecular detection of tuberculosis and rifampin resistance. *N Engl J Med*. 2010. doi:10.1056/NEJMoa0907847

5. Boehme CC, Nicol MP, Nabeta P, et al. Feasibility, diagnostic accuracy, and effectiveness of decentralised use of the Xpert MTB/RIF test for diagnosis of tuberculosis and multidrug resistance: A multicentre implementation study. *Lancet*. 2011. doi:10.1016/S0140-6736(11)60438-8

6. Steingart KR, Schiller I, Horne DJ, Pai M, Boehme CC, Dendukuri N. Xpert® MTB/RIF assay for pulmonary tuberculosis and rifampicin resistance in adults. *Cochrane Database Syst Rev*. 2014. doi:10.1002/14651858.CD009593.pub3

7. WHO. *Xpert MTB/RIF Assay for the Diagnosis of Pulmonary and Extrapulmonary TB in Adults and Children Policy Update*.; 2013.

8. Chakravorty S, Simmons AM, Rowneki M, et al. The new Xpert MTB/RIF ultra: Improving detection of Mycobacterium tuberculosis and resistance to Rifampin in an assay suitable for point-of-care testing. *MBio*. 2017. doi:10.1128/mBio.00812-17

9. Dorman SE, Armstrong DT, King B, et al. Xpert MTB/RIF Ultra for detection of Mycobacterium tuberculosis and rifampicin resistance: a prospective multicentre diagnostic accuracy study. *Lancet Infect Dis*. 2018.

10. Albert H, Nathavitharana RR, Isaacs C, Pai M, Denkinger CM, Boehme CC. Development, roll-out and impact of Xpert MTB/RIF for tuberculosis: What lessons have we learnt and how can we do better? *Eur Respir J*. 2016. doi:10.1183/13993003.00543-2016

14 Appendices

Appendix 1: Protocol Amendment Summary Table

Appendix 2: Package Insert for MMQCI Controls

Appendix 1: Protocol Amendment Summary Table

The Protocol Amendment Summary of Changes Table for the current amendment is located directly before the Abbreviations section.

Amendment [amendment number]: *([date])*

Overall Rationale for the Amendment

Section # and Name	Description of Change	Brief Rationale

Appendix 2: Package Insert for MMQCI Controls

Research Use Only

Maine Molecular Quality Controls, Inc.
23 Mill Brook Road, Saco, ME 04072 USA
Phone: 207-885-1072, FAX: 207-885-1079
Web: www.mmqci.com, Email: info@mmqci.com

INTROL® TB PANEL M114*plus*
INSTRUCTIONS FOR USE

INTENDED USE:

INTROL® TB Panel M114*plus* is intended for use as a quality control to monitor the nucleic acid detection of *M. tuberculosis* (MTB) and the mutations associated with multi-drug resistant MTB (MDR-TB) on the GeneXpert® System.

The World Health Organization (WHO) reports that about 1.7 billion people, 23% of the world's population, are estimated to have a latent TB infection, and are thus at risk of developing active TB disease during their lifetime.[1] There has been major progress in subsequent years – more than 60 million people have been documented as treated and cured since 2000, and case and death rates have fallen steadily. Nevertheless, worldwide, around 10 million people still fall ill with the disease each year (more adults than children, and more men than women), and TB is one of the top 10 causes of death. It is also the leading cause of death from a single infectious agent, ranking above HIV/AIDS. [2]

INTROL® TB Panel M114*plus* is provided for Research Use Only (RUO). It cannot be cloned, sold, or transferred without the explicit written consent of MMQCI.

PRODUCT SUMMARY and PRINCIPLE:

INTROL® TB Panel M114*plus* is composed of 3 positive controls and 1 negative control. The positive controls consist of non-infectious, synthetic MTB DNA encapsulated in bacterial cells. The MTB DNA does not include the entire MTB genome. MTB gene segments present among the positive controls are: IS6110, IS1081, hsp65, 16S rRNA, 23S rRNA, inhA, katG, and rpoB. Drug resistance mutations are incorporated in segments inhA, katG, and rpoB, as indicated in Table 1. Not all mutations listed are detected by Xpert MTB assays on the GeneXpert System. The negative control contains buffer and preservative only.

Best practice is to establish a quality control program for every assay performed by the laboratory.[3, 4] Routine use of quality controls that are consistent lot to lot and monitor the entire assay assists the laboratory in identifying shifts, trends, and increased frequency of random errors caused by variations in the test system, such as failing reagents and pipetting errors. Early investigation can prevent failed assay runs.

COMPOSITION:

INTROL® TB Panel M114*plus* is comprised of three bottles each of INTROL® TBWT-04, INTROL® TBMDR1-04, INTROL® TB1081MDR2, and INTROL® TBNEG, 1.0 mL each. INTROL® TBWT-04 and INTROL® TBMDR1-04 contain the following MTB gene segments: IS6110, hsp65, 16S rRNA, 23S rRNA, inhA, katG, and rpoB. INTROL® TB1081MDR2 contains MTB gene segments: IS1081, hsp65, 16S rRNA, 23S rRNA, inhA and rpoB. The MTB gene segments consist of non-infectious, synthetic MTB DNA encapsulated in chemically fixed and killed laboratory bacterial cells suspended in buffer and preservatives. INTROL® TBNEG contains buffer and preservative only. The presence or absence of drug resistance mutations is specified for each bottle in Table 1.

Table 1. Drug Resistance Mutations* found in INTROL® TB Panel M114*plus*

Control	Drug Resistance Mutation
INTROL® TBWT-04	no mutations/ wildtype (H37Rv)
INTROL® TBMDR1-04	rpoB: F505L, L511P, D516V, H526Y
	inhA: -15
	katG: S315T (AGC> ACC)
INTROL® TB1081MDR2	rpoB: S522L, H526D, S531L<br>inhA Mutant: -8T>C
INTROL® TBNEG	No MTB DNA, no cells

*References citing the mutations can be found at www.tbdreamdb.com

PRECAUTIONS AND WARNINGS:

INTROL® TB Panel M114*plus* has been tested on the GeneXpert® System only. It cannot be cloned, sold, or transferred without the explicit written consent of MMQCI. This product does not contain any biological material of human origin or infectious microorganisms. Do not freeze.

STORAGE and STABILITY:

Upon receipt and after opening, the material should be stored at 2° – 8°C. Do not freeze.
Unopened controls are stable through the expiration date printed on each bottle when stored at 2° – 8°C. Opened material tightly capped and returned to the refrigerator (2° – 8°C) shortly after use is stable for thirty (30) days from the date of opening.

INSTRUCTIONS FOR USE ON THE GeneXpert® System:

1. Each bottle contains 1.0 mL of control material.
2. Allow controls to come to room temperature.
3. Thoroughly mix controls by vigorously inverting several times immediately before use.
4. Before opening bottle, shake down or tap bottle on hard surface to be sure all liquid is out of cap.
5. Add 2.0 mL Sample Reagent to each control vial.
6. Mix by inverting the vial 10 times.
7. Let the vial sit at room temperature for 15 minutes. Invert several times half way through incubation period as you would for a sputum sample.
8. Open the Xpert® MTB/RIF cartridge lid and transfer 2.0 mL of respective Sample Reagent treated control, using a sterile transfer pipette. Close lid.
9. Transfer cartridge to the GeneXpert system.
10. Scan cartridge, enter sample ID and start the run.

LIMITATIONS:

INTROL® TB Panel M114*plus* is designed for use with MTB amplification assays that target one or more of the following MTB gene segments: IS6110, IS1081, hsp65, 16S rRNA, 23S rRNA, inhA, katG, and rpoB . Only those segments are present in INTROL® TB Panel M114*plus*.

EXPECTED VALUES:

Control	Xpert MTB/RIF<br>Expected result
INTROL® TBWT-04	MTB DETECTED<br>Rif Resistance NOT Detected
INTROL® TBMDR1-04	MTB DETECTED<br>Rif Resistance DETECTED
INTROL® TB1081MDR2	MTB DETECTED<br>Rif Resistance DETECTED
INTROL® TBNEG	MTB NOT DETECTED

REFERENCES:

1. WHO report 2018: https://www.who.int/tb/publications/global_report/en/
2. "Ten Facts About Tuberculosis," WHO, September 2018: http://www.who.int/features/factfiles/tuberculosis/en/index.html
3. ISO 15189: Medical laboratories – Particular requirements for quality and competence.
4. CAP Molecular Pathology Checklist; Commission on Laboratory Accreditation, Laboratory Accreditation Program, Mol.20000

ORDERING INFORMATION:

INTROL® TB Panel M114*plus*
Part Number: M114*plus*
Kit Contains: 12 bottles x 1.0mL
3 each TBWT-04, TBMDR1-04, TB1082MDR2, & TBNEG

M114*plus* 092319.000
