# Supplementary material for: Equivalence of the GeneXpert System and GeneXpert Omni System for tuberculosis and rifampicin resistance detection
Source: PLoS One. 2021 Dec 17;16(12):e0261442. doi: 10.1371/journal.pone.0261442 (PMC8682871; doi:10.1371/journal.pone.0261442)
Supplement: S1 Table — (DOCX) [file pone.0261442.s003.docx]

**S1 Table. Geographical spread and characteristics of tested specimens.**

| **Origin** | **Total number of specimens** | **Culture Negative** | **Culture Positive** | **Culture Positive (n=160)^b^** | | | | |
| --- | --- | --- | --- | --- | --- | --- | --- | --- |
|  |  |  |  | **Smear Negative** | **Smear Scanty** | **Smear 1+** | **Smear 2+** | **Smear 3+** |
| **Georgia** | **22** | **0** | **22** | 18 | 2 | 1 | 1 | 0 |
| **Italy** | **40** | **40** | **0** | 0 | 0 | 0 | 0 | 0 |
| **Moldova** | **75** | **0** | **75^a^** | 29 | 19 | 15 | 6 | 6 |
| **S Africa** | **1** | **0** | **1** | 1 | 0 | 0 | 0 | 0 |
| **Vietnam** | **62** | **0** | **62** | 5 | 13 | 12 | 8 | 24 |
| **Total** | **200/200 (100%)** | **40/200 (20%)** | **160/200 (80%)** | **53/160 (33%)** | **33/160 (21%)** | **28/160 (18%)** | **15/160 (9%)** | **30/160 (19%)** |

^a^ Of the 160 tuberculosis culture-positive specimens selected, one was excluded from the study due to insufficient volume for testing on both the Omni and GeneXpert devices.

^b^ Almost all tuberculosis culture-positive specimens (99%; 158/160) were rifampicin-resistant because availability of whole genome sequencing results was a requirement for specimen selection and sequencing was available mainly for specimens coming from FIND’s specimen collection of drug-resistant patients for whom rifampicin-resistance is an inclusion criterion.
