# Supplementary material for: Equivalence of the GeneXpert System and GeneXpert Omni System for tuberculosis and rifampicin resistance detection
Source: PLoS One. 2021 Dec 17;16(12):e0261442. doi: 10.1371/journal.pone.0261442 (PMC8682871; doi:10.1371/journal.pone.0261442)
Supplement: S2 Table — (DOCX) [file pone.0261442.s004.docx]

**S2 Table. Rifampicin resistance-associated mutations in tuberculosis-positive specimens tested in the Omni bioequivalence study.**

| **Drug of Interest** | **Reference genes** | **Mutations** | **Number of Specimens** |
| --- | --- | --- | --- |
| Rifampicin | *rpoB* | Wildtype | 5^a^ |
|  |  | Q432K | 2 |
|  |  | Q432L | 1 |
|  |  | Q432P | 2 |
|  |  | D435G, I491F | 1 |
|  |  | D435G, L452P | 1 |
|  |  | D435V | 12 |
|  |  | S441L | 1 |
|  |  | H445D | 7 |
|  |  | H445L | 1 |
|  |  | H445N | 3 |
|  |  | H445P | 1 |
|  |  | H445R | 3 |
|  |  | H445Y | 11 |
|  |  | L430P, D435G | 3 |
|  |  | S450F | 4 |
|  |  | S450L | 98 |
|  |  | S450W | 1 |
|  |  | L452P | 2 |
|  |  | Q429H | 1 |
|  |  | **TOTAL** | **160^b^** |

^a^ Three of the wildtype specimens with wildtype *rpoB* sequences were phenotypically rifampicin-resistant. A composite reference standard of both whole genome sequencing and phenotypic drug susceptibility testing was used in the analysis for this study.

^b^ Of the 160 tuberculosis culture-positive specimens, one (S450L mutant) was excluded from the study due to insufficient volume for testing on both the Omni and GeneXpert devices.
