# Supplementary material for: Equivalence of the GeneXpert System and GeneXpert Omni System for tuberculosis and rifampicin resistance detection
Source: PLoS One. 2021 Dec 17;16(12):e0261442. doi: 10.1371/journal.pone.0261442 (PMC8682871; doi:10.1371/journal.pone.0261442)
Supplement: S3 Table — (DOCX) [file pone.0261442.s005.docx]

**S3 Table. Listing of discordant rifampicin resistance detection results on Omni versus GeneXpert.**

| **Sample ID** | **Omni TB** | **Omni RIF** | **GX TB** | **GX RIF** | **pDST** | **WGS** | **Smear** |
| --- | --- | --- | --- | --- | --- | --- | --- |
| TB002133538 | TB+; Very low | RIF+ | TB+; Very low | RIF- | R | H445Y | Negative |
| TB002133610 | TB+; Low | RIF- | TB+; Low | RIF- | R | D435G & I491F | Positive (scanty) |
| TB002133646 | TB+; Medium | RIF+ | TB+; High | RIF Indeterminate | R | Q432L & K446E | Positive (2+) |
| TB002210050 | TB+; Very low | RIF+ | TB+; Trace | RIF Indeterminate | R | S450L | Negative |
| TB002133547 | TB+; High | RIF- | TB+; High | RIF Indeterminate | R | Q432P | Positive (2+) |
| TB002133582 | TB+; High | RIF- | TB+; High | RIF Indeterminate | R | Q432P | Positive (3+) |
| TB002210062 | TB+; High | RIF Indeterminate | TB+; High | RIF+ | R | L430P & D435G | Negative |
| TB002210003 | TB+; Trace | RIF Indeterminate | TB+; Low | RIF+ | R | S450L | Negative |
| TB002133716 | TB+; Medium | RIF Indeterminate | TB+; Medium | RIF- | R | wildtype | Positive (2+) |
| TB002133552 | TB+; High | RIF Indeterminate | TB+; High | RIF Indeterminate | R | Q429H | Positive (3+) |
| TB002220003 | TB+; Trace | RIF Indeterminate | TB+; Trace | RIF Indeterminate | R | S450L | Negative |
| TB002210063 | TB+; Medium | RIF- | TB+; Medium | RIF+ | S | wildtype | Negative |

ID; identification, GX; GeneXpert, pDST; phenotypic drug susceptibility test, R; resistant, RIF; rifampicin call, S; sensitive, TB; tuberculosis, WGS; whole genome sequencing

Truly discordant results (i.e. rifampicin-call not concordant with the reference standard) are highlighted in red font.
