# Supplementary material for: Equivalence of the GeneXpert System and GeneXpert Omni System for tuberculosis and rifampicin resistance detection
Source: PLoS One. 2021 Dec 17;16(12):e0261442. doi: 10.1371/journal.pone.0261442 (PMC8682871; doi:10.1371/journal.pone.0261442)
Supplement: S4 Table — (DOCX) [file pone.0261442.s006.docx]

**S4 Table. Equivalence of Xpert Ultra Cts and Tms comparing Omni to GeneXpert at normal environmental conditions.**

| **Parameter** | **Probe** | **MMQCI Control** | **Estimate [90% CI]** | **Equivalence assessment [TOST approach]** |
| --- | --- | --- | --- | --- |
| Ct | SPC | TBNEG | -0.60 [ -1.80, 0.60] | **Equivalence shown** *[CI is within: -1.9 and +1.9]* |
|  |  | TB-WT | -0.41 [ -0.74, -0.07] | **Equivalence shown** *[CI is within: -1.7 and +1.7]* |
|  |  | TB-MDR2 | -0.36 [ -0.70, -0.02] | **Equivalence shown** *[CI is within: -2.1 and +2.1]* |
|  | IS1081-IS6110 | TB-WT | -1.05 [ -1.42, -0.69] | **Equivalence shown** *[CI is within: -2.4 and +2.4]* |
|  |  | TB-MDR2 | -0.27 [ -0.66, 0.11] | **Equivalence shown** *[CI is within: -2.4 and +2.4]* |
|  | rpoB1 | TB-WT | -1.86 [ -2.28, -1.44] | **Equivalence shown** *[CI is within: -2.9 and +2.9]* |
|  |  | TB-MDR2 | -0.80 [ -1.19, -0.41] | **Equivalence shown** *[CI is within: -2.7 and +2.7]* |
|  | rpoB2 | TB-WT | -1.88 [ -2.30, -1.46] | **Equivalence shown** *[CI is within: -2.9 and +2.9]* |
|  |  | TB-MDR2 | -0.83 [ -1.23, -0.42] | **Equivalence shown** *[CI is within: -2.9 and +2.9]* |
|  | rpoB3 | TB-WT | -2.23 [ -2.69, -1.77] | **Equivalence shown** *[CI is within: -2.9 and +2.9]* |
|  | rpoB4 | TB-WT | -1.89 [ -2.39, -1.38] | **Equivalence shown** *[CI is within: -2.9 and +2.9]* |
|  |  | TB-MDR2 | -1.11 [ -1.49, -0.73] | **Equivalence shown** *[CI is within: -2.9 and +2.9]* |
| Tm | rpoB1 | TB-WT | -0.45 [ -0.51, -0.39] | **Equivalence shown** *[CI is within: -1 and +1]* |
|  |  | TB-MDR2 | -0.47 [ -0.53, -0.41] | **Equivalence shown** *[CI is within: -1 and +1]* |
|  | rpoB2 | TB-WT | -0.42 [ -0.48, -0.36] | **Equivalence shown** *[CI is within: -1 and +1]* |
|  | rpoB3 | TB-WT | -0.33 [ -0.39, -0.27] | **Equivalence shown** *[CI is within: -1 and +1]* |
|  | rpoB4 | TB-WT | -0.27 [ -0.33, -0.20] | **Equivalence shown** *[CI is within: -1 and +1]* |
| Mutant Tm | rpoB2 | TB-MDR2 | -0.33 [ -0.39, -0.27] | **Equivalence shown** *[CI is within: -1 and +1]* |
|  | rpoB4 | TB-MDR2 | -0.54 [ -0.60, -0.49] | **Equivalence shown** *[CI is within: -1 and +1]* |

CI; confidence interval, Ct; cycle threshold, MDR; multidrug-resistant, MMQCI; Maine Molecular Quality Controls, SPC; sample processing control, Tm; melting temperature, TOST; two one-sided tests, WT; wild type

Equivalence limits were set a priori based upon data provided by the manufacturer regarding the standard deviation in values for 5 Ultra cartridge lots. a TOST (two one-sided test) was used as a test of equivalence  to test the hypothesis of equality between two means. Estimates are computed as Ct/Tm(GeneXpert) – Ct/Tm(Omni), such that negative values indicate increased Ct/Tm-values on Omni.
