# Supplementary material for: Equivalence of the GeneXpert System and GeneXpert Omni System for tuberculosis and rifampicin resistance detection
Source: PLoS One. 2021 Dec 17;16(12):e0261442. doi: 10.1371/journal.pone.0261442 (PMC8682871; doi:10.1371/journal.pone.0261442)
Supplement: S5 Table — (DOCX) [file pone.0261442.s007.docx]

**S5 Table. Equivalence of Xpert Ultra Cts and Tms comparing Omni at challenging environmental conditions to GeneXpert at normal environmental conditions.**

| **Parameter** | **Probe** | **MMQCI Control** | **Estimate** | **Equivalence assessment** |
| --- | --- | --- | --- | --- |
|  |  |  | **[90% CI]** | **[TOST approach]** |
| Ct | SPC | TBNEG | -0.2 | **Equivalence shown** |
|  |  |  | [ -1.58, 1.18] | *[CI is within: -1.9 and +1.9]* |
|  |  | TB-WT | 0.04 | **Equivalence shown** |
|  |  |  | [ -0.25, 0.33] | *[CI is within: -1.7 and +1.7]* |
|  |  | TB-MDR2 | -0.13 | **Equivalence shown** |
|  |  |  | [ -0.44, 0.18] | *[CI is within: -2.1 and +2.1]* |
|  | IS1081-IS6110 | TB-WT | -0.32 | **Equivalence shown** |
|  |  |  | [ -0.74, 0.11] | *[CI is within: -2.4 and +2.4]* |
|  |  | TB-MDR2 | 0.12 | **Equivalence shown** |
|  |  |  | [ -0.24, 0.49] | *[CI is within: -2.4 and +2.4]* |
|  | rpoB1 | TB-WT | -0.47 | **Equivalence shown** |
|  |  |  | [ -0.94, -0.01] | *[CI is within: -2.9 and +2.9]* |
|  |  | TB-MDR2 | 0.19 | **Equivalence shown** |
|  |  |  | [ -0.19, 0.58] | *[CI is within: -2.7 and +2.7]* |
|  | rpoB2 | TB-WT | -0.55 | **Equivalence shown** |
|  |  |  | [ -1.03, -0.07] | *[CI is within: -2.9 and +2.9]* |
|  |  | TB-MDR2 | 0.16 | **Equivalence shown** |
|  |  |  | [ -0.24, 0.55] | *[CI is within: -2.9 and +2.9]* |
|  | rpoB3 | TB-WT | -0.51 | **Equivalence shown** |
|  |  |  | [ -1.01, -0.01] | *[CI is within: -2.9 and +2.9]* |
|  | rpoB4 | TB-WT | -0.73 | **Equivalence shown** |
|  |  |  | [ -1.37, -0.10] | *[CI is within: -2.9 and +2.9]* |
|  |  | TB-MDR2 | 0.2 | **Equivalence shown** |
|  |  |  | [ -0.21, 0.61] | *[CI is within: -2.9 and +2.9]* |
| Tm | rpoB1 | TB-WT | -0.13 | **Equivalence shown** |
|  |  |  | [ -0.23, -0.04] | *[CI is within: -1 and +1]* |
|  |  | TB-MDR2 | -0.13 | **Equivalence shown** |
|  |  |  | [ -0.24, -0.03] | *[CI is within: -1 and +1]* |
|  | rpoB2 | TB-WT | -0.09 | **Equivalence shown** |
|  |  |  | [ -0.17, -0.00] | *[CI is within: -1 and +1]* |
|  | rpoB3 | TB-WT | -0.08 | **Equivalence shown** |
|  |  |  | [ -0.16, -0.00] | *[CI is within: -1 and +1]* |
|  | rpoB4 | TB-WT | -0.11 | **Equivalence shown** |
|  |  |  | [ -0.21, -0.02] | *[CI is within: -1 and +1]* |
| Mutant Tm | rpoB2 | TB-MDR2 | -0.06 | **Equivalence shown** |
|  |  |  | [ -0.16, 0.05] | *[CI is within: -1 and +1]* |
|  | rpoB4 | TB-MDR2 | -0.2 | **Equivalence shown** |
|  |  |  | [ -0.28, -0.12] | *[CI is within: -1 and +1]* |

CI; confidence interval, Ct; cycle threshold, MDR; multidrug-resistant, MMQCI; Maine Molecular Quality Controls, SPC; sample processing control, Tm; melting temperature, TOST; two one-sided tests, WT; wild type

Equivalence limits were set a priori based upon data provided by the manufacturer regarding the standard deviation in values for 5 Ultra cartridge lots. a TOST (two one-sided test) was used as a test of equivalence  to test the hypothesis of equality between two means. Estimates are computed as Ct/Tm(GeneXpert) – Ct/Tm(Omni), such that negative values indicate increased Ct/Tm-values on Omni.
